# Hyperscanning of Interactive Juggling: Expertise Influence on Source Level Functional Connectivity

**DOI:** 10.3389/fnhum.2019.00321

**Published:** 2019-09-18

**Authors:** David B. Stone, Gabriella Tamburro, Edson Filho, Selenia di Fronso, Claudio Robazza, Maurizio Bertollo, Silvia Comani

**Affiliations:** ^1^BIND – Behavioral Imaging and Neural Dynamics Center, University “G. d’Annunzio” of Chieti-Pescara, Chieti, Italy; ^2^Department of Neuroscience, Imaging and Clinical Sciences, University “G. d’Annunzio” of Chieti-Pescara, Chieti, Italy; ^3^SINAPSE – Social Interaction and Performance Science Laboratory, School of Psychology, University of Central Lancashire, Preston, United Kingdom; ^4^Department of Medicine and Aging Sciences, University “G. d’Annunzio” of Chieti-Pescara, Chieti, Italy

**Keywords:** hyperscanning, EEG, interactive juggling, graph theory, skill-level

## Abstract

Hyperscanning studies, wherein brain activity is recorded from multiple participants simultaneously, offer an opportunity to investigate interpersonal dynamics during interactive tasks at the neurophysiological level. In this study, we employed a dyadic juggling paradigm and electroencephalography (EEG) hyperscanning to evaluate functional connectivity between EEG sources within and between jugglers’ brains during individual and interactive juggling. We applied graph theoretical measures to identify significant differences in functional connectivity between the individual and interactive juggling conditions. Connectivity was measured in multiple juggler pairs with various skill levels where dyads were either skill-level matched or skill-level unmatched. We observed that global efficiency was reduced during paired juggling for less skilled jugglers and increased for more skilled jugglers. When jugglers were skill-level matched, additional reductions were found in the mean clustering coefficient and small-world topology during interactive juggling. A significant difference in hemispheric brain lateralization was detected between skill-level matched and skill-level unmatched jugglers during interactive juggling: matched jugglers had an increased right hemisphere lateralization while unmatched jugglers had an increased left hemisphere lateralization. These results reveal multiple differences in functional brain networks during individual and interactive juggling and suggest that similarities and disparities in individual skills can impact inter-brain dynamics in the performance and learning of motor tasks.

## Introduction

Juggling is a unique and complex skill that depends on multiple perceptual-motor and cognitive abilities and processes including coordination, visuo-spatial attention, motor vigilance, performance related self-monitoring, and sensorimotor learning (Sánchez García et al., 2013; Rodrigues et al., 2016). Advances in neuroimaging, which permit the acquisition of brain data in ecological settings during active movement (Thompson et al., 2008), have allowed opportunities to study these processes at the neurophysiological level. As a consequence, juggling has been used to investigate skill acquisition, motor learning, and expert performance by examining movement related cortical potentials and functional and structural brain connectivity (Gerber et al., 2014; Schiavone et al., 2015; Berchicci et al., 2017).

Juggling can be performed individually and between two or more interactive participants. Thus, it represents an opportunity to explore social factors such as team dynamics, cooperation, and interpersonal interaction (Filho et al., 2015). By acquiring neuroimaging data from multiple participants simultaneously (i.e., via hyperscanning) these factors can be investigated also at the neurophysiological level.

In the last several years, hyperscanning techniques have expanded the field of social neuroscience and are yielding new insights into the neurodynamics that underlie interpersonal interaction (for reviews, see Babiloni and Astolfi, 2014; Liu et al., 2018; Wang et al., 2018; Zhang, 2018). By recording brain activity from two or more participants simultaneously using electroencephalography (EEG), near infrared spectroscopy (NIRS), or functional magnetic resonance imaging (fMRI), hyperscanning allows researchers to examine *hyperbrain* functional networks, which include the functional connectivity within the brain of each participant (intra-brain connectivity) as well as the functional connectivity between the brains of two or more participants (inter-brain connectivity).

We recently applied EEG hyperscanning to explore functional connectivity in the emergent hyperbrain network of two jugglers engaged in interactive (dyadic) juggling (Filho et al., 2016). In this proof of concept work, we paired one expert juggler with a novice juggler and examined hyperbrain connectivity in dyadic juggling tasks of increasing difficulty. We identified unique patterns of inter-brain connectivity as well as several differences between the intra-brain functional networks of the two jugglers as task demands increased, suggesting that skill-level (e.g., expert vs. novice) may influence hyperbrain dynamics. Previous evidence supports differences in functional brain connectivity between expert and non-expert jugglers during individual juggling tasks (Schiavone et al., 2015), and some hyperscanning studies have examined hyperbrain connectivity between two experts (e.g., Sänger et al., 2012; Toppi et al., 2016). However, to date the effects of skill-level on hyperbrain dynamics during interpersonal tasks have not been systematically explored.

In the present study, we sought to extend our previous work by examining interactive juggling in multiple pairs of jugglers with diverse levels of expertise. To better assess the influence of skill-level on hyperbrain dynamics, we quantified general properties of hyperbrain functional networks at source level using measures derived from graph theory and measures of regional and hemispheric connectivity. Specifically, the aims of the present study were to (a) quantify differences in functional connectivity during individual juggling versus dyadic juggling tasks, (b) examine how individual juggling skill might affect functional connectivity in both conditions, and (c) explore how similar or dissimilar skill-levels affect functional connectivity in juggling dyads.

## Materials and Methods

To meet the aforementioned aims, we acquired EEG data from multiple pairs of jugglers during both individual and interactive juggling, reconstructed hyperbrain functional connectivity maps at the source level, and employed multiple measures of functional brain network topology to typify functional connectivity within and between jugglers’ brains.

### Study Participants

Thirteen jugglers (12 males; aged 25.3 ± 4.4 years), recruited from a professional juggling school in Northern Italy, participated in the study. Jugglers had between 3 and 19 years of experience in individual juggling but no prior experience in interactive juggling. All jugglers provided informed written consent prior to study participation according to the policies outlined in the Declaration of Helsinki and approved by the local institutional ethics committee.

### Experimental Design

The experimental task was based on previous research on the juggling paradigm. The juggling paradigm purports that interactive juggling serves as an ideal platform to study social interaction in general, and the notion of team mental models in particular (Filho et al., 2015, 2016, 2017). Specifically, Filho et al. (2015) have shown that interactive juggling prevents social loafing, as performance depends on all interacting partners, and allows for the examination of how individuals’ characteristics (e.g., skill level) and task constraints (e.g., increase in the number of balls to be juggled) influence psycho-bio-social variables. In the present study, we were interested in evaluating the role of skill level on within- and between-brains functional connectivity. Seven separate juggling sessions comprised the study. Each session consisted of a unique pairing of two jugglers. One juggler (Subj. 10, left handed) participated in two separate sessions conducted on two separate days. The remaining jugglers participated in only one session. In each session, the jugglers had either a similar or a dissimilar skill-level, quantified with their number of years of juggling experience. Based on the distribution of the years of experience difference between jugglers in a dyad, we defined a MATCHED skill-level group comprising three sessions with jugglers’ experience difference ≤ 3 years, and an UNMATCHED skill-level group comprising four sessions with jugglers’ experience difference > 3.

**FIGURE 1:**
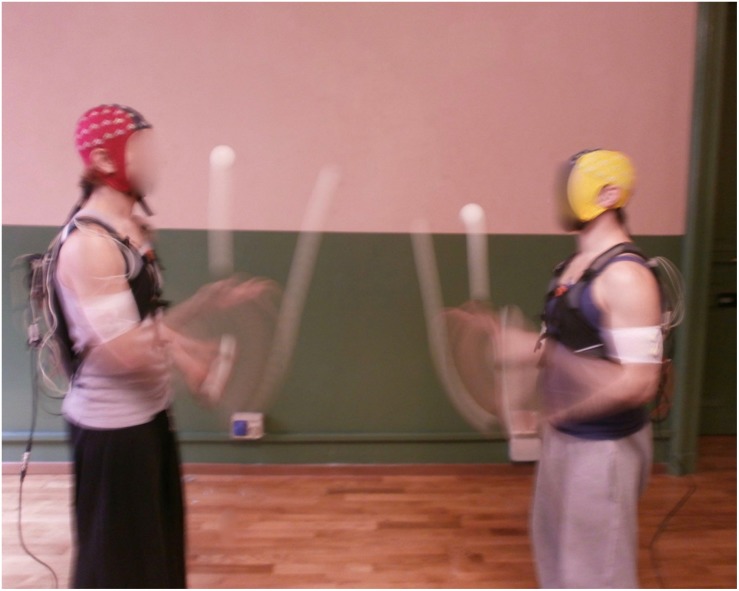
Jugglers during the EEG acquisition in the PAIRED condition. Written informed consent for the publication of this image was obtained from the individuals appearing in it.

In each session, data were collected from two jugglers in two conditions: an individual juggling (SOLO) condition and a paired juggling (PAIRED) condition. Both conditions consisted of juggling trials of at least 20 s duration with an inter–trial interval of approximately 1 min to allow the jugglers restart when they felt ready. In the SOLO trials, each juggler was asked to juggle three balls continuously in a so-called *half-shower* pattern. In the PAIRED trials, the same two jugglers were asked to juggle five balls between them (interactively) in an adapted half-shower pattern (Figure 1). Importantly, as our goal was to evaluate (within and between brains) functional connectivity during interactive motor actions rather than to experimentally manipulate social variables, during the interactive condition the participants were not instructed to mimic, copy, observe, or lead or follow one another, nor were they given any motivational or performance feedback throughout the trials. The participants were only instructed to “keep the balls in the air for 20 s or as long as they could.” Also noteworthy, the different number of balls used in the SOLO and PAIRED conditions aimed at reproducing the same difficulty level in the two conditions, akin to a degrees of freedom *(df)* rationale (Filho et al., 2015) in which the *df* equals the total number of balls being juggled minus the number of hands involved in the task (2 for the SOLO and 4 for the PAIRED condition). If the jugglers failed to complete a 20 s trial in either condition, additional trials were completed until a total of 12 successful trials were obtained for each condition. The order of conditions was counter-balanced to control for learning and vigilance confounds.

### Electrophysiological Recording

**FIGURE 2:**
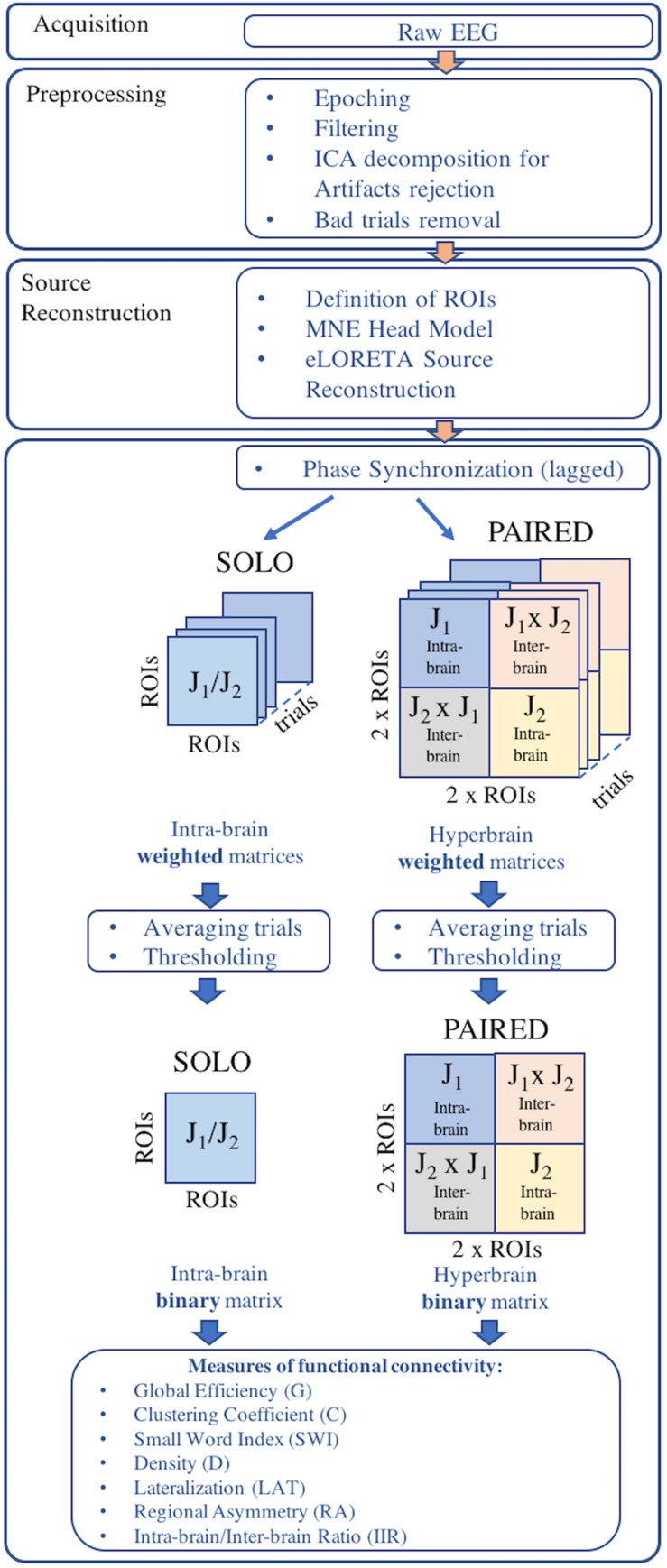
Overview of the EEG data processing pipeline.

An overview of the EEG data processing pipeline is provided in Figure 2. EEG data were acquired using two synchronized mobile EEG systems designed for acquisitions during sports applications (eegosports, ANT Neuro B.V., Enschede, Netherlands). Both systems employed unipolar biosignal amplifiers and 32 channel caps with Ag/AgCl electrodes arranged in a standard 10/5 montage (Oostenveld and Praamstra, 2001). Data were collected at a sampling frequency of 1024 Hz with a common average reference, where an additional Ag/AgCl electrode over the mastoid served as ground. Impedance values were verified prior to each acquisition and maintained less than 10 kΩ by adjusting electrode-to-scalp contacts. For the SOLO condition, the 12 trials were collected during a single EEG acquisition from each juggler independently. For the PAIRED condition, the 12 trials were collected in a single EEG acquisition from both jugglers simultaneously. In both conditions, a push-button trigger signal was sent to the EEG system(s) to demark the beginning and end of each trial and to synchronize the EEG acquisitions (during PAIRED condition).

### EEG Data Preprocessing

Electroencephalography recordings from each session were epoched into trials for each juggler in each condition. Only trials longer than or equal to 20 s (successful trials) were retained for further processing. Epoched trials were trimmed to 10 s by removing the first and last 5 s from each trial, thus retaining only the 10 s interval of steady juggling. All trials were band-pass filtered between 1 and 100 Hz and a notch filter was applied to remove power line interference at 50 Hz. The EEG time-series from each trial was visually inspected and EEG channels exhibiting excessive noise or absence of signal were removed.

To remove artifacts, each EEG trial was decomposed into 20 independent components (ICs) using an extended version of the Infomax ICA algorithm, which has been demonstrated to be more suitable to separate sources which may possess super-Gaussian and sub-Gaussian distributions (Lee et al., 1999). The topography, time course, and spectral power of each IC were visually inspected and the ICs of likely artifactual origin were removed. EEG trial time-series were then reconstructed from the retained ICs. Reconstructed time-series were re-inspected and the trials still exhibiting artifactual contamination were removed from further analysis. As a result, the total number of retained EEG trials available for the SOLO and PAIRED conditions varied across sessions. Participant information and descriptions of retained juggling sessions are presented in Table 1. All EEG pre-processing was performed in the Matlab environment (release R2018b; MathWorks, Natick, MA, United States) using functions from the EEGLAB toolbox (release 14.1.1b; Delorme and Makeig, 2004).

**TABLE 1:** Details on the retained juggling sessions.

**Session**	**Juggler**	**Subject**	**Dominance**	**Years of experience**	**Matched dyad**	**SOLO trials**	**PAIRED trials**
1	J1	Subj. 4	right	16	YES	8	4
	J2	Subj. 3	right	19		9	4
2	J1	Subj. 6	right	3	NO	9	3
	J2	Subj. 5	right	7		12	3
3	J1	Subj. 7	right	3	NO	9	6
	J2	Subj. 8	right	7		10	6
4	J1	Subj. 10	left	8	YES	8	6
	J2	Subj. 9	right	10		12	6
5	J1	Subj. 11	right	5	YES	10	4
	J2	Subj. 12	right	8		10	4
6	J1	Subj. 2	Right	6	NO	7	3
	J2	Subj. 13	Right	15		10	3
7	J1	Subj. 10	Left	8	NO	9	8
	J2	Subj. 1	left/right	14		8	8

*From left to right: juggling session, dyad composition (jugglers), jugglers’ ID, dominant hand, years of juggling experience; type of dyad (MATCHED or UNMATCHED), number of retained trials for each juggler from each recording condition (SOLO and PAIRED). Only one juggler was female, and she was also the only left-handed juggler (Subj. 10).*

### EEG Source Reconstruction

Potential cerebral sources of EEG activity from each retained trial were estimated using the exact LORETA (eLORETA) algorithm (Pascual-Marqui, 2007a) and implemented using the LORETA-KEY software^1^. The eLORETA algorithm constructs an inverse (source) solution based on a realistic head model employing the MNI152 template where the solution space is restricted to 6239 voxels of 5 mm^3^ resolution comprising possible cortical gray matter and hippocampal sources estimated from the probabilistic Talairach atlas (Lancaster et al., 2000). We confined our analysis of EEG sources to 13 single-voxel cortical regions of interest (ROIs) from each cerebral hemisphere for a total of 26 ROIs from each juggler’s brain (Table 2). Since we anticipated that juggling would likely engage multiple cortical networks, ROIs were selected to provide broad coverage of cortex while distances between sources were maintained to avoid potential over-estimates in connectivity between neighboring sources. We obtained the three-dimensional electrical current source densities (i.e., the current density in each of the three spatial dimensions) at each of the 26 ROIs for each juggler in each SOLO and PAIRED trial from all sessions.

**TABLE 2:** List of the 26 ROIs used for each juggler’s brain.

**Label**	**Extended Label**	**BA type**	**Hemisphere**	**ROI centroid MNI coordinates**		
				***x***	***y***	***z***
SMA	SensoriMotor Area	3	left	−40	−25	50
			right	40	−25	50
SPL	Superior Parietal Lobule	7	left	−15	−60	50
			right	15	−60	50
SPFC	Superior PreFrontal Cortex	6	left	−25	0	50
			right	25	0	50
OFC	OrbitoFrontal Cortex	11	left	−25	35	−15
			right	25	35	−15
APFC	Anterior PreFrontal Cortex	10	left	−25	45	25
			right	25	45	25
LPFC	Lateral PreFrontal Cortex	45	left	−50	20	15
			right	50	20	15
ACC	Anterior Cingulate Cortex	24	left	−5	20	25
			right	5	20	25
PCC	Posterior Cingulate Cortex	30	left	−5	−50	20
			right	5	−50	20
PHG	ParaHippocampal Gyrus	28	left	−20	−20	−20
			right	20	−20	−20
IPC	Inferior Parietal Cortex	40	left	−45	−45	35
			right	45	−45	35
FUS	Fusiform cortex	37	left	−40	−65	0
			right	40	−65	0
PVC	Primary Visual Cortex	17	left	−15	−85	0
			right	15	−85	0
INS	INSULA	13	left	−40	−10	10
			right	40	−10	10

*From left to right: Labels, Extended labels, Broadmann areas, Hemisphere, MNI coordinates along the *x*, *y*, and *z* axes.*

### EEG Functional Connectivity Estimation

We chose to investigate functional connectivity only in the alpha frequency band (8.5–12 Hz) because (1) alpha band frequencies have been shown to be an important marker of human social coordinated behavior (Tognoli et al., 2007) and (2) our previous research identified differences in hyperbrain functional connectivity between expert and non-expert jugglers at alpha band frequencies (Filho et al., 2017).

To estimate functional connectivity within and between the jugglers’ brains, we calculated the phase synchronization (PS) between all ROIs within each juggler’s brain in the SOLO and PAIRED conditions and between jugglers’ brains in the PAIRED condition for each retained trial. PS is a measure of stability of phase difference between two time-series (i.e., the phase difference between the electrical current source densities at each ROI). PS values vary between 0 and 1, where zero indicates random phase differences and unity indicates a constant phase difference between two time-series. Because PS can over-estimate the degree of connectivity between brain sources due to contamination from brain volume conduction effects (Nolte et al., 2004), we considered only the lagged component of phase synchronization (LPS) between sources, which ignores the instantaneous component likely due to volume conduction (Pascual-Marqui, 2007b). We calculated the LPS for hyperbrain matrices as well to maintain a common single measure of connectivity, although connectivity between sources from two different brains would not be contaminated by volume conduction artifacts. The calculation of LPS in both SOLO and PAIRED conditions provided the further advantage that we could use a common threshold for all connectivity maps (see below) and therefore directly compare intra-brain and inter-brain connectivity.

To calculate the LPS between ROI pairs, we employed a method described by Pascual-Marqui (2007c) which is suitable for computing synchronizations between pairs of time series in three spatial dimensions (i.e., between the time series of the three-dimensional current source densities of each ROI pair). The method proposed by Pascual-Marqui (2007c) is briefly described herein. For each ROI, the 10 s time courses of the three-dimensional source current densities were segmented into segments of 2 s duration and 0.5 s overlap, for a total of nine segments for each trial. Then, the 2 s segments were transformed into the frequency domain by applying a fast Fourier transform, resulting in a 3-by-1 complex-valued vector at every 0.5 Hz frequency from 0 to 512 Hz for each segment. To preserve only the phase, these vectors were normalized by factoring out the amplitudes at each frequency. For each ROI and each segment of 2 s duration, the complex-valued auto-spectral (normalized) covariance matrices were then calculated at each frequency by multiplying each 3-by-1 vector by its complex conjugate. Additionally, the complex-valued cross-spectral (normalized) covariance matrices were calculated for each pair of ROIs and each 2 s segment at each frequency bin by multiplying the 3-by-1 vector from one ROI by the complex conjugate of the other ROI in the pair. At this point, the auto-spectral and cross-spectral covariance matrices were averaged across all nine 2 s segments for each trial and for all ROI pairs and used to calculate the LPS at each frequency bin between all ROI pairs. The LPS values for each ROI pair from each 0.5 Hz frequency bin between 8.5 and 12 Hz were then averaged to obtain the final mean LPS for that ROI pair in the 8.5–12 Hz frequency band. All original code for the calculation of LPS intra-brain and inter-brain connectivity is publicly available at https://doi.org/10.6084/m9.figshare.9384512.

The preceding procedure was applied between all ROI pairs for each trial. In the SOLO condition, where 26 ROIs were defined, each ROI was compared to the remaining 25 ROIs. Because PS is a bi-directional measure, this resulted in 325 (i.e., [(26 × 25)/2]) intra-brain averaged LPS values for each SOLO condition trial. In the PAIRED condition, there were 26 ROIs from each of the two jugglers’ brains. Thus, there were 325 intra-brain ROI pairs for each juggler and an additional 676 (i.e., 26^2^) inter-brain ROI pairs, resulting in a total of 1326 averaged LPS values for each PAIRED condition trial.

A thresholding procedure was applied to each set independently to transform the LPS matrices into binary adjacency matrices. The median of the averaged LPS and the median average deviation were calculated for each set. A threshold was applied to each set such that averaged LPS values greater than or equal to the median plus the median average deviation were set to 1; otherwise, the averaged LPS values were set to 0 (Li et al., 2015; Chella et al., 2016).

**FIGURE 3:**
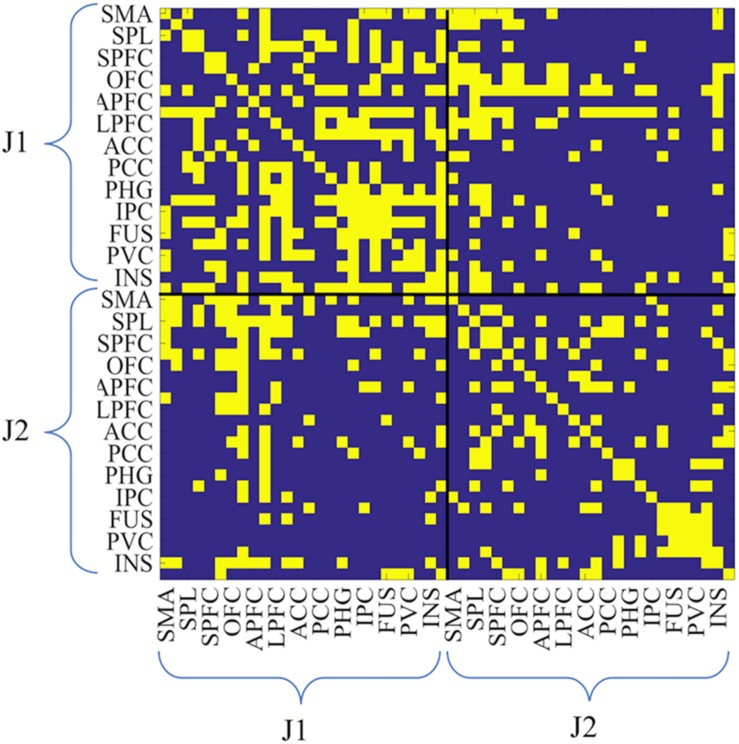
Example of hyperbrain binary adjacency matrix (session 3, and PAIRED condition). This matrix includes 52-by-52 ROIs: the upper left and the lower right matrices are the intra-brain connectivity maps of J1 and J2, whereas the upper right and the lower left matrices are the inter-brain connectivity maps (**upper right**: J1 × J2; **lower left**: J2 × J1). As for all matrices, J1 is the less experienced juggler. In the case reported in this figure J1 had 3 years of juggling experience and J2 had 7 years of juggling experience. Connectivity values were coded as zero if below the threshold, as unity if above the threshold.

Thus, for each session, there were two 26-by-26 ROI intra-brain adjacency matrices for the SOLO condition (one from each juggler) where the diagonals were self-connections. Additionally, there was one 52-by-52 ROI adjacency matrix for the PAIRED condition (hyperbrain matrix) where the upper and lower diagonal matrices were intra-brain connectivity maps and the off diagonal matrices were inter-brain connectivity maps (Figure 3). In each dyad, J1 was always less experienced than J2, regardless of the absolute difference in their years of juggling experience.

### Global Measures of Functional Connectivity

To synthetically describe the features of the functional networks (intra-brain, inter-brain, and hyperbrain connectivity maps), we employed multiple measures of functional brain network topology, including global measures derived from Graph Theory, which provides effective means to quantify the degree of integration, segregation, and efficiency in functional brain networks (Watts and Strogatz, 1998; Rubinov and Sporns, 2010). We also employed measures of regional asymmetry and hemispheric lateralization to better describe hyperbrain connectivity. The list of measures calculated for each type of functional connectivity map (intra-brain, inter-brain, and hyperbrain) is given in Table 3.

**TABLE 3:** Functional connectivity measures.

**Measures of functional connectivity**	**Intra-brain matrices**	**Inter-brain matrices**	**Hyperbrain matrices**
Global Efficiency (G)	✓		✓
Clustering Coefficient (C)	✓		✓
Small Word Index (SWI)	✓		✓
Density (D)	✓		✓
Lateralization (LAT)	✓	✓	
Regional Asymmetry (RA)	✓	✓	
Intra-brain/Inter-brain Ratio (IIR)			✓

*List of functional connectivity measures calculated for the binary intra-brain, inter-brain and hyperbrain matrices.*

#### Measures of Functional Efficiency

*Global efficiency* (*G*) is a graph theoretic measure which quantifies the ability of a network to exchange information globally and is generally defined as the reciprocal or inverse of the average path length (*L*) of the network (Bullmore and Sporns, 2009). The latter, in turn, is defined as the average number of steps along the shortest paths for all possible pairs of network nodes. Therefore, *L* is a measure of the efficiency of information transport in a functional network.

To provide information about the extent to which a network incorporates totally disordered (random) or totally regular (lattice) structural features, *L* is normalized by comparing the characteristic path length obtained in the observed network to the characteristic path lengths expected in reference networks with random and/or lattice configurations (Sporns and Zwi, 2004). Random networks are characterized by short average path lengths and thus possess high global efficiency whereas lattice networks are characterized by longer path lengths and possess low global efficiency. Therefore, for a given network to possess significant global efficiency its values of *L* are expected to deviate from the value of *L* observed in lattice networks and approach the values observed in random networks.

To estimate the global efficiency of the intra-brain and hyperbrain networks during the SOLO and PAIRED juggling conditions, we calculated a measure of *G* suggested by Sporns and Zwi (2004), which is the inverse of the normalized average path length *L* calculated using 100 random networks and 100 lattice networks. The random and lattice networks were generated by changing the connectivity values between the ROIs in the original intra-brain or hyperbrain network while preserving the same intra-brain and inter-brain degree distributions as in the original network (Humphries and Gurney, 2008). Values of *G* vary between 0 and 1; these two extremes, respectively, correspond to the low global efficiency of a totally regular (or lattice) configuration (i.e., *G* = 0) and to the high global efficiency of a totally disordered (random) configuration (i.e., *G* = 1). Because it is possible for *G* to occasionally be less than 0 or greater than 1, particularly in highly sparse or dense networks, values less than 0 were set to 0 and values greater than 1 were set to 1 (Muldoon et al., 2016).

The *clustering coefficient* (*C*) is a measure of the prevalence of clustered connectivity around individual nodes (ROIs) and reflects the tendency of the network to exchange information locally (Watts and Strogatz, 1998). Unlike global efficiency, high values of *C* indicate that a high number of connections exist among neighboring nodes. Therefore, random networks are characterized by low values of *C* and lattice networks are characterized by high values of *C*. Thus, for a given network to possess a significant degree of clustering, its value of *C* is expected to deviate from the *C* observed in random networks and approach values observed in lattice networks. We calculated C according to the normalization method proposed by Sporns and Zwi (2004) and employing the same normalization procedure used for the global efficiency *G*. We then obtained *normalized C* values that could vary between 0 and 1, respectively, corresponding to the low local information exchange of a random configuration and to the high local information exchange of a lattice configuration. If obtained values of *C* were greater than 1 or less than 0, *C* was set to 1 or 0, respectively.

The *small world index* (*SWI*) is a measure of a network’s “small-worldness,” which is a tendency for a functional network to jointly possess a short average path length and a high mean average clustering coefficient as compared to random and lattice networks. Small-world networks therefore have the unique feature of combining high global and local efficiencies. It has been demonstrated that the functional organization of the human brain has small-world features both at rest and during the execution of simple motor tasks (Bassett et al., 2006), meaning that the human brain exhibits an optimal balance of functional integration and segregation (Sporns et al., 2000; Rubinov and Sporns, 2010), is highly efficient at both local and global levels, and is therefore more capable of adapting to changing task demands (Bassett et al., 2006).

The “small-worldness” of a functional network is generally quantified with a metric introduced by Humphries and Gurney (2008), which is a function of both the mean and normalized *C* and the mean and normalized *L* of the network. More recently, Neal (2017) introduced a “small-world” index (*SWI*) that aims to measure the extent to which the two small-world characteristics of an observed network are jointly maximized, and can be calculated as the product of the normalized global efficiency *G* and the normalized clustering coefficient *C.* Given that both *G* and *C* can range from 0 to 1, also the product of these two terms (i.e., *SWI*) ranges from 0 to 1: when the network displays only one or neither small-world characteristic (i.e., when either *G* or *C* or both equal 0), *SWI* equals 0; when the network displays both small-world characteristics (i.e., when both *G* and *C* equal 1), *SWI* equals 1.

All calculations of *L* and *C* and the generation of random and lattice networks were performed using the Brain Connectivity Toolbox (Rubinov and Sporns, 2010).

#### Measures of Connectivity Extent

*Connection density* (*D*) is another global measure from Graph Theory that describes the extent of connectivity in a network. *D* is defined as the proportion of connections out of all possible connections in graph, and is the simplest estimator of the physical cost (e.g., the energy or other resource requirements) of a network (Bullmore and Sporns, 2009; Bassett and Bullmore, 2017). *D* is calculated as the actual number of edges (connections) in the graph as a proportion of the total number of possible edges, which equals 325 for intra-brain networks, and 1326 for hyperbrain networks.

The *intra-brain/inter-brain ratio* (*IIR*) is a measure unique to hyperbrain networks that quantifies the asymmetry in the functional brain maps of interacting subjects. *IIR* is defined as the ratio between the total number of intra-brain connections from both jugglers in the network and the number of inter-brain connections between the jugglers (Toppi et al., 2015).

#### Measures of Lateralization and Regional Asymmetry

Based on prior functional mapping studies where intrinsic brain activity lateralization within the attention system in normal subjects (Fox et al., 2006) and the memory system in patients (Wang et al., 2009) were observed, we built *lateralization* (*Lat*) and *regional asymmetry* (*RA*) measures similar to those used in other reports (Jansen et al., 2006; Seghier, 2008; Liu et al., 2009) to quantify regional or hemispheric dominance in the functional connectivity maps. *Lat* and *RA* describe how intra-brain and inter-brain connections are distributed in the network based on the number of connections within or between specific brain regions (partial sets of ROIs).

For intra-brain networks, *Lat*_*intra*_ quantifies the asymmetry between the number of connections within the left and right hemispheres of each juggler. The values of *Lat*_*intra*_ are bounded between –1 and +1, where +1 indicates that all observed intra-brain connections occur in the right hemisphere, –1 indicates that all observed intra-brain connections occur in the left hemisphere, and 0 indicates that there are an equal number of right and left intra-brain connections (i.e., no lateralization of connectivity). *RA*_*intra*_ quantifies asymmetries between fronto-limbic regions and occipito-parietal regions. The values of *RA*_*intra*_ are likewise bounded between +1 (only fronto-limbic intra-brain connections) and –1 (only occipito-parietal intra-brain connections) with a value near 0 indicating lack of regional asymmetry.

For inter-brain networks, *Lat* and *RA* are calculated for the connections between jugglers’ brains. In this case, *Lat*_*inter*_ and *RA*_*inter*_ are measures specific to each juggler’s brain in the hyperbrain network and describe asymmetries in the distribution of connections to/from the left and right hemispheres and to/from the fronto-limbic and occipito-parietal regions of the brain of each juggler during dyadic juggling. As with the intra-brain connections, values of *Lat*_*inter*_ and *RA*_*inter*_ are bounded between –1 and +1.

### Data Analysis

In order to accomplish the aims of the study, we first performed a series ANOVAs where we compared the intra-brain networks of each of the two jugglers from each session during the SOLO and PAIRED conditions. In each session, one of the jugglers had at least two more years of juggling experience than the other, allowing us to include juggling skill (defined as years of juggling experience) as a factor in the ANOVAs by comparing the intra-brain network of the more skilled juggler with the intra-brain network of the less skilled juggler. Thus, we performed three-factor ANOVA tests where skill-level (more vs. less skilled) and condition (SOLO vs. PAIRED) were within-session factors, and similarity in jugglers’ skill-levels (MATCHED vs. UNMATCHED) was a between-session factor. We performed six separate ANOVAs to identify significant differences in each of the different measures of functional connectivity applicable to intra-brain networks: *G*, *C*, *SWI*, *D*, *Lat*_*intra*_, and *RA*_*intra*_. We then evaluated functional connectivity in the MATCHED only sessions and UNMATCHED only sessions separately by performing two-factor ANOVAs for each skill-level group (MATCHED or UNMATCHED) where skill-level (more vs. less skilled) and condition (SOLO vs. PAIRED) were within-session factors. To determine the direction of effects, follow-up *t*-tests were performed for all ANOVAs that reached significance (*p* = 0.05). These follow-up tests were Bonferroni corrected to account for multiple comparisons.

We also investigated differences in inter-brain connectivity (i.e., *Lat*_*inter*_ and *RA*_*inter*_) due to skill-level (more vs. less skilled) and similarity of jugglers’ skill-levels (MATCHED vs. UNMATCHED) by performing two-factor ANOVAs in the PAIRED condition.

Finally, we tested for differences in functional connectivity in the hyperbrain networks as a whole during PAIRED juggling due to similarity of skill-level. We performed five independent *t*-tests to compare *G*, *C*, *SWI*, *D*, and *IIR* between hyperbrain networks in the MATCHED and UNMATCHED skill-level groups.

## Results

The first aim of the study was to quantify the differences in functional connectivity patterns during individual juggling and dyadic juggling tasks.

At the intra-brain level, the three-factor ANOVA tests performed for the applicable measures of functional connectivity (i.e., *G*, *C*, *SWI*, *D*, *Lat*_*intra*_, and *RA*_*intra*_) revealed significant differences between the SOLO and PAIRED conditions in global efficiency (*G*) and hemispheric lateralization (*Lat*_*intra*_).

**FIGURE 4:**
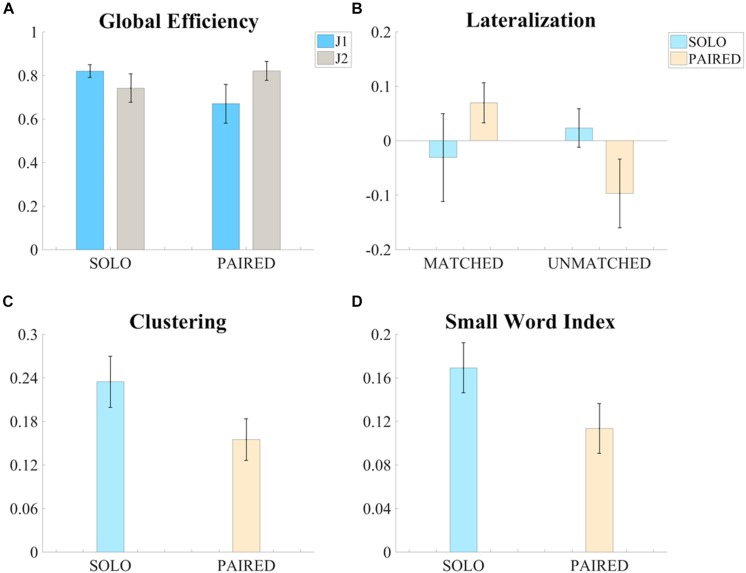
Significant results of the ANOVA tests on the measures of functional connectivity. **(A)** Bar graph of the global efficiency (G – Mean ± SEM) of the intra-brain adjacency matrices for the SOLO and PAIRED conditions and for the two skill levels (J1 always less skilled than J2). **(B)** Bar graph of the Lateralization (Lat – Mean ± SEM) values of intra-brain adjacency matrices of the SOLO and PAIRED conditions for the MATCHED and UNMATCHED skill level groups. **(C)** Bar graph of the clustering coefficient (C – Mean ± SEM) values of the intra-brain adjacency matrices (MATCHED group only) for the SOLO and PAIRED conditions. **(D)** Bar graph of the Small Word Index (SWI – Mean ± SEM) of the intra-brain adjacency matrices (MATCHED group only) for the SOLO and PAIRED conditions

**FIGURE 5:**
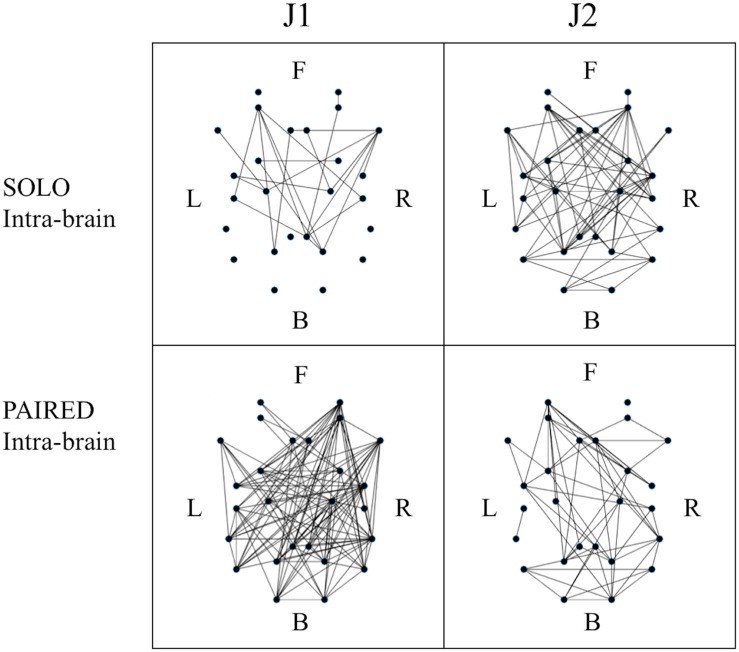
Example of binary intra-brain functional connectivity graphs for the SOLO and PAIRED juggling conditions. All graphs refer to session 4 which is a MATCHED session (J1 = Subj. 10; J2 = Subj. 9). The dots in each graph represent the 26 ROIs as listed in Table 2 in relative 2D coordinates. Anterior (Frontal – F) sources are represented at the **top**; posterior (Back – B) sources are represented at the **bottom**; **left** hemisphere sources (Left – L) are to the left of each graph; **right** hemisphere sources (Right – R) are to the right of each graph.

The three-factor ANOVA test of global efficiency (*G*) revealed a significant condition (SOLO vs. PAIRED) by skill-level (more vs. less skilled) interaction such that the less skilled juggler showed a decrease in *G* whereas the more skilled juggler showed an increase in *G* during PAIRED juggling compared to the SOLO juggling condition [*F*(1,5) = 8.392, *p* = 0.034, η_*p*_^2^ = 0.627, Power = 0.643; Figure 4A). The effect was greater for the less skilled juggler, approaching significance [*t*(6) = 2.233, *p* = 0.067, Cohen *d* = 0.844]. No other significant contrasts were observed. Results for all comparisons are provided in the Supplementary Table S1. Examples of intra-brain functional connectivity maps for the SOLO and PAIRED juggling conditions are shown in Figure 5.

The results on intra-brain global efficiency also confirmed that juggling experience affects functional connectivity in individual and dyadic juggling (second aim of the study). The effect of skill-level on the connectivity maps of dyadic juggling was further explored with the three-factor ANOVA test of intra-brain hemispheric lateralization (*Lat*_*intra*_), which revealed a significant condition (SOLO vs. PAIRED) by skill-level groups (MATCHED vs. UNMATCHED) interaction [*F*(1,5) = 8.376, *p* = 0.034, η_*p*_^2^ = 0.626, Power = 0.642; Figure 4B). While follow-up contrasts did not reveal significant differences, a shift to a right lateralized intra-brain functional connectivity in the PAIRED condition compared to the SOLO condition was observed in MATCHED jugglers, while there was a shift to left lateralization in UNMATCHED jugglers. Results for all comparisons are provided in the Supplementary Table S1.

To further address the third aim of the study, we assessed how similarities or differences in jugglers’ skill-level within a dyad (i.e., MATCHED or UNMATCHED skill-level groups) affected functional connectivity during the dyadic juggling task. We found significant effects only for MATCHED jugglers: For this group, there was a significant reduction in clustering (*C*) and small-worldness (*SWI*) during PAIRED juggling compared to SOLO juggling [*C*: *F*(1,2) = 19.751, *p* = 0.047, η_*p*_^2^ = 0.908, Power = 0.637; Figure 4C; *SWI*: *F*(1,2) = 21.413, *p* = 0.044, η_*p*_^2^ = 0.915, Power = 0.666; Figure 4D].

No significant differences in inter-brain connectivity (*Lat*_*inter*_ and *RA*_*inter*_) were found, although there was a trend for the less skilled juggler to possess right lateralized inter-brain connectivity while the more skilled juggler possessed greater left lateralized inter-brain connectivity in both skill-level groups (MATCHED and UNMATCHED; *F*(1,1) = 5.557, *p* = 0.065, η_*p*_^2^ = 0.527, Power = 0.479). See Supplementary Table S2 for results of all ANOVA tests performed.

**FIGURE 6:**
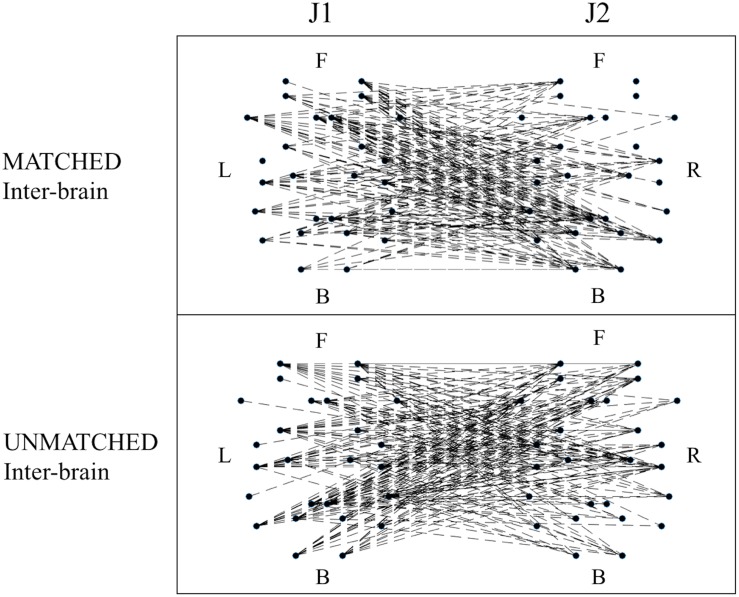
Examples of functional connectivity in the inter-brain graph from a MATCHED (session 1, J1 = Subj. 4, J2 = Subj. 3) and an UNMATCHED (session 3, J1 = Subj. 7, J2 = Subj. 8) juggling session. In each graph, the dots represent the 52 ROIs as listed in Table 2; of these dots, the 26 dots on the **left** represent the ROIs of J1’s brain, and the labels L, F, and B indicate the Left, Frontal and Back areas of J1’s brain; the other 26 dots on the **right** represent the ROIs of J2’s brain, and the labels R, F, and B indicate the Right, Frontal and Back areas of J2’s brain.

When examining differences in functional connectivity in the hyperbrain maps from MATCHED juggling sessions compared to the hyperbrain maps from UNMATCHED juggling sessions, no significant differences were found for any measure of functional connectivity (Figure 6 and Supplementary Table S3).

## Discussion

The aims of the present study were to quantify differences in functional connectivity during individual and interactive juggling, assess how individual skill-level affected these differences, and explore how similarities/disparities in skill-level affected connectivity during paired juggling. We found multiple significant differences in functional connectivity between individual and interactive juggling, including differences in global efficiency, which depended upon individual skill-level, and hemispheric lateralization, which depended upon similarities and differences in skill-level. Additional differences were found only when interactive jugglers possessed similar juggling skills, including differences in clustering and small-worldness. These results demonstrate that individual and interactive juggling elicit distinct patterns of functional connectivity and that these differences are mediated by juggling skill.

Overall, our results show a general reduction in functional connectivity during PAIRED juggling, including reduced global efficiency for the less skilled juggler and reduced clustering and small-world organization for both jugglers in matched-skill dyads. This reduced functional connectivity may have occurred because interactive juggling was a more difficult task than individual juggling. In our previous research, we manipulated task difficulty during paired juggling by increasing the number of balls juggled. Results indicated that there was a decrease in functional connectivity as task difficulty increased, reflected in reduced clustering, increased path lengths, and reduced small-world organization (Filho et al., 2016). These differences are similar to the differences observed here between SOLO and PAIRED juggling conditions. However, it remains unclear whether these differences are due solely to increased task demands or if they reflect other underlying differences between individual and interactive juggling. Including an additional dimension of task difficulty during both SOLO and PAIRED juggling conditions will allow us to specifically identify how task demand contributes to changes in functional brain connectivity. This analysis is planned in future research.

An alternative explanation for the reduced functional connectivity observed is that interactive juggling may have induced new motor learning since participants possessed little or no previous interactive juggling experience. Research suggests that the effects of motor learning on functional brain organization are complex, particularly in the acquisition of juggling skills. Sampaio-Baptista et al. (2015) reported that solo juggling training in novices resulted in either increased or decreased functional connectivity in motor networks depending on the intensity of training sessions, such that high intensity training (30 min/day) increased functional connectivity and low intensity training (15 min/day) decreased it. The PAIRED condition in our study could represent a low intensity training session. However, these authors only reported differences in functional connectivity after a 6-week training period. De Vico Fallani et al. (2010) specifically evaluated changes in functional connectivity during different phases in the acquisition of a new visuo-motor task. These authors found an increase in global efficiency in alpha band connectivity during the initial phase of visuo-motor learning, followed by a decline to baseline during subsequent phases. In the present study, we evaluated functional connectivity in the SOLO and PAIRED conditions for a single session averaged across multiple trials. In future studies, we may examine differences in connectivity between single trials during familiar (SOLO juggling) and unfamiliar (PAIRED juggling) conditions to specifically determine how functional brain organization changes during different learning phases.

We found that PAIRED juggling elicited greater intra-brain lateralization compared to SOLO juggling; however, the direction of lateralization depended on whether jugglers were skill-matched: Jugglers with similar skill-levels showed increased right intra-brain lateralization when juggling together, while jugglers with disparate skill-levels showed increased left intra-brain lateralization. Lateralization during paired juggling may reflect increased activation in hemisphere specific visuo-spatial or motor attentional networks (Heilman and Van Den Abell, 1980; Rushworth et al., 2001) or activation of lateralized motor control and adaptation systems (Mutha et al., 2012). Further, these networks may be preferentially activated by the particular demands of juggling with someone with similar or superior/inferior skills, which likely evoke different shared and complementary mental models (Filho et al., 2017) as well as unique leader-follower dynamics (Sänger et al., 2013). In particular, the coordination of interactive motor tasks hinges on the activation of shared and complementary networks within and between the brains of the interacting individuals (Filho et al., 2017). Skill-level may also modulate leader-follower dynamics insofar that less-skilled individuals follow more skilled individuals who, in turn, are more likely to initiate action (Dumas et al., 2012; Sänger et al., 2013).

Several differences observed during interactive juggling occurred only when jugglers were skill-matched. To our knowledge, there have been no previous reports that have specifically addressed how similarities or disparities in participants’ skill-level affect functional brain organization during interactive tasks, although there are several reports of increased functional connectivity during cooperative tasks performed by expert pairs (e.g., Sänger et al., 2012; Toppi et al., 2016). Due to difficulties in recruiting professional jugglers with diverse juggling skills, capable of performing the demanding interactive juggling task, we were limited to only a few cases where jugglers were matched on skill-level during PAIRED juggling. The small number of cases in our study may explain why we did not find significant differences in hyperbrain functional organization between skill-level matched and unmatched interactive juggling. We reiterate that our sample size estimation was not probabilistic in nature but rather constrained to our access to skilled jugglers. As detailed elsewhere, the study of skilled performers in a naturalistic environment is a trade-off between high ecological validity and sample size (Filho et al., 2015). The magnitude effect size and observed power metrics reported throughout the manuscript attest to the robustness of our findings and are akin to the current standards of reporting set by the American Psychological Association (Appelbaum et al., 2018). Specifically, our results suggest that skill-level similarity/disparity is an important axis to consider when designing experiments that probe functional brain organization during interactive participation and warrants further investigation in its own right.

The reliability of our results is supported by the fact that we calculated hyperbrain functional connectivity at the source level, as done previously in very few EEG studies (Astolfi et al., 2011; Toppi et al., 2015). By using source level analysis and by selecting a broad set of ROIs to characterize functional connectivity across a wide-spread cortical network, we overcame a common limitation of many EEG hyperbrain studies where the use of sensor level analysis can obscure accurate identification of the brain areas involved in functional networks (Nunez and Srinivasan, 2006; Schoffelen and Gross, 2009).

In the current study, we limited our functional connectivity analysis to the alpha frequency band because it has been shown to be an important marker of human social coordinated behavior (Tognoli et al., 2007) and because our previous research identified differences in hyperbrain functional connectivity between expert and non-expert jugglers at alpha band frequencies (Filho et al., 2017). Nevertheless, additional differences in functional connectivity are likely to occur at other frequency bands (Schiavone et al., 2015). Examining functional connectivity in other bands and directly comparing functional networks across bands could yield further insights into differences between individual and interactive juggling.

An additional limitation of the present study is that we treated phase synchronization as a binary variable by applying a thresholding procedure; however, modeling brain connectivity as a binary network likely represents an over-simplification (Bassett and Bullmore, 2017). In the present work, our purpose was to identify, describe, and compare functional networks in individual and interactive juggling. In follow-up studies, we will apply measures of effective connectivity to uncover patterns of directionality and determine how these patterns evolve during interactive juggling between jugglers with various skill-levels, extending our analysis to other frequency bands and including psychological factors.

To conclude, despite the aforementioned limitations, our study clearly revealed that multiple differences in functional brain networks during individual and interactive juggling exist, thus suggesting that individuals’ skill levels influence inter-brain dynamics in the performance and potentially the learning of complex motor tasks. Perhaps most importantly, our study is among the first to estimate source level connectivity across brains engaged in a naturalistic task and thus advances our neuropsychological understanding of the costs and benefits of teamwork performed by teammates with different skill levels, while also advancing a stepwise methodology [see attached homemade scripts available on FigShare (https://doi.org/10.6084/m9.figshare.9384512)] to model complex brain dynamics during complex “real-world” interactive tasks.

## Data Availability

The raw data supporting the conclusions of this manuscript will be made available by the authors, without undue reservation, to any qualified researcher.

## Ethics Statement

This study was carried out in accordance with the recommendations of the Guidelines for Sperimentazione clinica non farmacologica no profit, monocentrica, University G. d’Annunzio of Chieti-Pescara (Italy). This protocol was approved by the University G. d’Annunzio of Chieti-Pescara (Italy) with Ethical Application Ref. no. 10-21/05/2015. All subjects gave written informed consent in accordance with the Declaration of Helsinki.

## Author Contributions

All co-authors contributed to the study design. EF, SdF, and MB collected the data. DS, GT, and SC designed the procedure for data analysis. DS developed the software and wrote the manuscript. DS and GT analyzed the data. SC supervised all phases of the work. All co-authors evaluated the results and revised the manuscript.

## Conflict of Interest Statement

The authors declare that the research was conducted in the absence of any commercial or financial relationships that could be construed as a potential conflict of interest.

## Footnotes

1 http://www.uzh.ch/keyinst/loreta.htm
