# Supplementary material for: Hyperscanning of Interactive Juggling: Expertise Influence on Source Level Functional Connectivity
Source: Front Hum Neurosci. 2019 Sep 18;13:321. doi: 10.3389/fnhum.2019.00321 (PMC6760461; doi:10.3389/fnhum.2019.00321)
Supplement: Supplementary file 1 [file Table_1.pdf]

Supplementary Table S1.

Statistical comparison of Global efficiency in the SOLO and PAIRED conditions. Statistical results of follow-up contrasts directly comparing the values of the intra-brain global efficiency in the SOLO and PAIRED conditions for J1 (lower skill level) and J2 (higher skill level). From left to right: first sample (sample 1), number of subjects in first sample (n_sample1), second sample (sample 2), numbers of subjects in second sample (n_sample2), t-test value (t), degrees of freedom (df), p value (p), Cohen's d value for paired samples Cohen d.

	**sample 1**	**n_sample1**	**sample 2**	**n_sample2**	**t**	**df**	**p**	**Cohen d**
**Global efficiency**	SOLO J1	7	SOLO J2	7	0.977	6	0.366	0.369
	PAIRED J1	7	PAIRED J2	7	1.485	6	0.188	0.561
	SOLO J1	7	PAIRED J1	7	2.233	6	0.067	0.844
	SOLO J2	7	PAIRED J2	7	0.784	6	0.463	0.296

Statistical results of follow-up contrasts directly comparing the values of the intra-brain LAT in the SOLO and PAIRED conditions for MATCHED and UNMATCHED experience. From left to right: first sample (sample 1), number of subjects in first sample (n_sample1), second sample (sample 2), numbers of subjects in second sample (n_sample2), t-test value (t), degrees of freedom (df), p value (p), Cohen's d value for independent samples in the first and second cell Cohen d , Cohen's d value for paired samples in the third and fourth cell Cohen d.

	**sample 1**	**n_sample1**	**sample 2**	**n_sample2**	**t**	**df**	**p**	**Cohen d**
**LAT *intra-brain***	SOLO MATCHED	3	SOLO UN-MATCHED	4	0.684	5	0.524	0.522
	PAIRED MATCHED	3	PAIRED UN-MATCHED	4	2.058	5	0.095	1.571
	SOLO MATCHED	3	PAIRED MATCHED	3	2.288	2	0.149	1.320
	SOLO UN-MATCHED	4	PAIRED UN-MATCHED	4	2.128	3	0.123	1.064
