# Supplementary material for: Hyperscanning of Interactive Juggling: Expertise Influence on Source Level Functional Connectivity
Source: Front Hum Neurosci. 2019 Sep 18;13:321. doi: 10.3389/fnhum.2019.00321 (PMC6760461; doi:10.3389/fnhum.2019.00321)
Supplement: Supplementary file 2 [file Table_2.pdf]

Supplementary Table S2. Detailed results of ANOVA tests.

Three-factor ANOVA with 1 between-session factors (matched vs. unmatched) and 2 within-session factors (condition; SOLO vs. PAIRED) x (experience; less-skilled vs. more-skilled) (*p < 0.05)

Factors	Measure	df1	df2	F	p	η_p^2	Power
condition	G	1	5	0.218	0.660	0.042	0.067
	C	1	5	0.317	0.598	0.060	0.075
	W	1	5	1.787	0.239	0.263	0.194
	D	1	5	1.383	0.293	0.217	0.161
	LAT	1	5	0.066	0.807	0.013	0.055
	RA	1	5	0.231	0.651	0.044	0.068
condition * match	G	1	5	0.004	0.953	0.001	0.050
	C	1	5	1.401	0.290	0.219	0.163
	SW	1	5	1.496	0.276	0.230	0.170
	D	1	5	1.764	0.242	0.261	0.192
	LAT	1	5	8.376	0.034*	0.626	0.642
	RA	1	5	1.604	0.261	0.243	0.179
experience	G	1	5	0.367	0.571	0.068	0.079
	C	1	5	0.525	0.501	0.095	0.092
	SW	1	5	0.062	0.813	0.012	0.055
	D	1	5	0.003	0.961	0.001	0.050
	LAT	1	5	0.718	0.436	0.126	0.107
	RA	1	5	0.657	0.454	0.116	0.102
experience * match	G	1	5	0.926	0.380	0.156	0.124
	C	1	5	0.982	0.367	0.164	0.129
	SW	1	5	0.510	0.507	0.092	0.090
	D	1	5	0.104	0.761	0.020	0.058
	LAT	1	5	0.058	0.819	0.011	0.055
	RA	1	5	0.708	0.438	0.124	0.106
condition * experience	G	1	5	8.392	0.034*	0.627	0.643
	C	1	5	0.197	0.676	0.038	0.065
	SW	1	5	1.084	0.346	0.178	0.137
	D	1	5	2.455	0.178	0.329	0.248
	LAT	1	5	0.103	0.761	0.020	0.058
	RA	1	5	0.764	0.422	0.132	0.111
condition * experience * match	G	1	5	3.258	0.131	0.395	0.312
	C	1	5	0.194	0.678	0.037	0.065
	SW	1	5	0.318	0.597	0.060	0.075
	D	1	5	1.622	0.259	0.245	0.181
	LAT	1	5	2.241	0.195	0.310	0.231
	RA	1	5	0.044	0.842	0.009	0.053

match	G	1	5	1.845	0.232	0.270	0.199
	C	1	5	0.544	0.494	0.098	0.093
	SW	1	5	1.089	0.345	0.179	0.137
	D	1	5	0.838	0.402	0.144	0.117
	LAT	1	5	0.633	0.462	0.112	0.100
	RA	1	5	0.722	0.434	0.126	0.107

Two-Factor RM-ANOVA with 2 factors (condition) × (experience), performed separately for the two dyad types UNMATCHED and MATCHED (*p < 0.05)

MATCHED	Factors	Measure	df1	df2	F	p	η_p^2	Power
UN-MATCHED ONLY	condition	G	1	2	0.083	0.792	0.027	0.055
		C	1	2	0.139	0.734	0.044	0.058
		SW	1	2	0.005	0.949	0.002	0.050
		D	1	2	2.784	0.194	0.481	0.219
		LAT	1	2	4.529	0.123	0.602	0.318
		RA	1	2	0.789	0.440	0.208	0.098
	experience	G	1	2	0.116	0.756	0.037	0.057
		C	1	2	1.614	0.294	0.350	0.149
		SW	1	2	0.553	0.511	0.156	0.084
		D	1	2	0.031	0.872	0.010	0.052
		LAT	1	2	0.560	0.509	0.157	0.084
		RA	1	2	0.000	0.986	0.000	0.050
	condition * experience	G	1	2	0.462	0.546	0.133	0.078
		C	1	2	0.669	0.473	0.182	0.091
		SW	1	2	2.028	0.250	0.403	0.174
		D	1	2	0.032	0.869	0.011	0.052
		LAT	1	2	0.648	0.480	0.178	0.090
		RA	1	2	0.287	0.629	0.087	0.068
MATCHED ONLY	condition	G	1	2	0.158	0.730	0.073	0.057
		C	1	2	19.751	0.047*	0.908	0.637
		SW	1	2	21.413	0.044*	0.915	0.666
		D	1	2	0.019	0.903	0.009	0.051
		LAT	1	2	5.237	0.149	0.724	0.264
		RA	1	2	0.736	0.481	0.269	0.083
	experience	G	1	2	0.697	0.492	0.258	0.082
		C	1	2	0.034	0.870	0.017	0.052
		SW	1	2	0.091	0.791	0.044	0.054
		D	1	2	0.143	0.742	0.067	0.057
		LAT	1	2	0.269	0.656	0.119	0.062

		RA	1	2	15.401	0.059	0.885	0.552
	condition * experience	G	1	2	40.222	0.024	0.953	0.866
		C	1	2	0.000	0.999	0.000	0.050
		SW	1	2	0.072	0.814	0.035	0.053
		D	1	2	20.768	0.045	0.912	0.655
		LAT	1	2	2.286	0.270	0.533	0.150
		RA	1	2	0.444	0.574	0.182	0.070

Two-Factor ANOVAs of *inter-brain connectivity* in the PAIRED condition only with 1 between-session (matched) and 1 within-session factor (experience)

**Factors**	**Measure**	**df1**	**df2**	**F**	**p**	η_p^2	**Power**
experience	LAT	1	5	5.577	0.065	0.527	0.479
	RA	1	5	2.024	0.214	0.288	0.213
experience * match	LAT	1	5	0.810	0.409	0.139	0.115
	RA	1	5	0.470	0.524	0.086	0.087
match	LAT	1	5	1.220	0.320	0.196	0.148
	RA	1	5	0.219	0.659	0.042	0.067
