# Supplementary material for: Hyperscanning of Interactive Juggling: Expertise Influence on Source Level Functional Connectivity
Source: Front Hum Neurosci. 2019 Sep 18;13:321. doi: 10.3389/fnhum.2019.00321 (PMC6760461; doi:10.3389/fnhum.2019.00321)
Supplement: Supplementary file 3 [file Table_3.pdf]

Supplementary Table S3. T test results on hyperbrain connectivity measures. Statistical results of independent samples t-tests assessing the six functional connectivity measures of the hyperbrain connectivity matrices from the two different types of paired sessions (MATCHED vs. UNMATCHED). From left to right: type of functional connectivity measures, mean and standard deviation of functional connectivity measures for the MATCHED and UNMATCHED skill level groups, t- test value, degrees of freedom, p value, Cohen's d value for independent samples.

Functional connectivity measures	MATCHED	UNMATCHED	t	df	p	Cohen d
**G**	0.414 ± 0.081	0.478 ± 0.113	0.835	5	0.442	0.631
**C**	0.350 ± 0.183	0.307 ± 0.179	0.319	5	0.762	0.238
**SW**	0.139 ± 0.061	0.145 ± 0.092	0.091	5	0.931	0.074
**D**	0.228 ± 0.009	0.243 ± 0.011	1.905	5	0.115	1.464
**IIR**	0.687 ± 0.562	0.385 ± 0.078	1.093	5	0.324	0.838
